# Pseudo Test-Retest Evaluation of Millimeter-Resolution Whole-Brain Dynamic Contrast-enhanced MRI in Patients with High-Grade Glioma

**DOI:** 10.1148/radiol.2021203628

**Published:** 2021-06-08

**Authors:** Yannick Bliesener, R. Marc Lebel, Jay Acharya, Richard Frayne, Krishna S. Nayak

**Affiliations:** From the Ming Hsieh Department of Electrical and Computer Engineering, Viterbi School of Engineering, University of Southern California, 3740 McClintock Ave, EEB 400, Los Angeles, CA 90089-2564 (Y.B., K.S.N.); GE Healthcare, Calgary, Canada (R.M.L.); Department of Radiology, University of Calgary, Calgary, Canada (R.M.L.); Seaman Family MR Research Centre, Foothills Hospital, Calgary, Canada (R.M.L., R.F.); Department of Radiology, Keck School of Medicine, University of Southern California, Los Angeles, Calif (J.A., K.S.N.); and Departments of Radiology and Clinical Neuroscience, Hotchkiss Brain Institute, University of Calgary, Calgary, Canada (R.F.).

## Abstract

**Background:**

Advances in sub-Nyquist–sampled dynamic contrast-enhanced (DCE) MRI enable monitoring of brain tumors with millimeter resolution and whole-brain coverage. Such undersampled quantitative methods need careful characterization regarding achievable test-retest reproducibility.

**Purpose:**

To demonstrate a fully automated high-resolution whole-brain DCE MRI pipeline with 30-fold sparse undersampling and estimate its reproducibility on the basis of reference regions of stable tissue types during multiple posttreatment time points by using longitudinal clinical images of high-grade glioma.

**Materials and Methods:**

Two methods for sub-Nyquist–sampled DCE MRI were extended with automatic estimation of vascular input functions. Continuously acquired three-dimensional k-space data with ramped-up flip angles were partitioned to yield high-resolution, whole-brain tracer kinetic parameter maps with matched precontrast-agent T1 and M_0_ maps. Reproducibility was estimated in a retrospective study in participants with high-grade glioma, who underwent three consecutive standard-of-care examinations between December 2016 and April 2019. Coefficients of variation and reproducibility coefficients were reported for histogram statistics of the tracer kinetic parameters plasma volume fraction and volume transfer constant (K^trans^) on five healthy tissue types.

**Results:**

The images from 13 participants (mean age ± standard deviation, 61 years ± 10; nine women) with high-grade glioma were evaluated. In healthy tissues, the protocol achieved a coefficient of variation less than 57% for median K^trans^, if K^trans^ was estimated consecutively. The maximum reproducibility coefficient for median K^trans^ was estimated to be at 0.06 min^–1^ for large or low-enhancing tissues and to be as high as 0.48 min^–1^ in smaller or strongly enhancing tissues.

**Conclusion:**

A fully automated, sparsely sampled DCE MRI reconstruction with patient-specific vascular input function offered high spatial and temporal resolution and whole-brain coverage; in healthy tissues, the protocol estimated median volume transfer constant with maximum reproducibility coefficient of 0.06 min^–1^ in large, low-enhancing tissue regions and maximum reproducibility coefficient of less than 0.48 min^–1^ in smaller or more strongly enhancing tissue regions.

Published under a CC BY 4.0 license.

*Online supplemental material is available for this article.*

See also the editorial by Lenkinski in this issue.

## Graphical abstract

**Figure:**
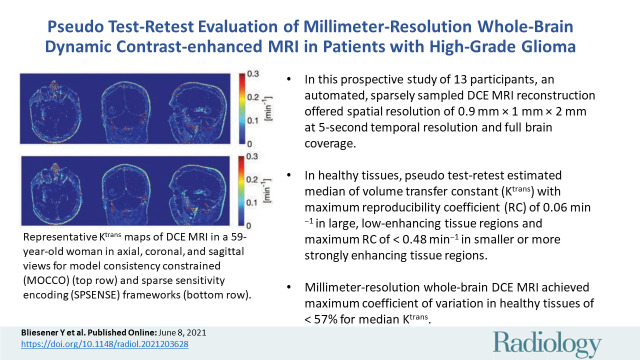

**Summary**

Evaluation of longitudinal millimeter-resolution whole-brain dynamic contrast-enhanced MRI bounds the coefficient of variation for median volume transfer constant to be less than 57% in healthy tissue regions.

**Key Results**

■ In this prospective study of 13 participants, an automated, sparsely sampled dynamic contrast-enhanced (DCE) MRI reconstruction offered spatial resolution of 0.9 mm × 1 mm × 2 mm at 5-second temporal resolution and full brain coverage.
■ In healthy tissues, pseudo test-retest in sparsely sampled DCE MRI estimated median of volume transfer constant (K^trans^) with maximum reproducibility coefficient (RC) of 0.06 min^–1^ in large, low-enhancing tissue regions and maximum RC of less than 0.48 min^–1^ in smaller or more strongly enhancing tissue regions.
■ Millimeter-resolution whole-brain DCE MRI achieved maximum coefficient of variation in healthy tissues of less than 57% for median K^trans^.

## Introduction

Dynamic contrast-enhanced (DCE) MRI enables assessment of neurovascular parameters by monitoring enhancement patterns in tissue after injection of contrast agent. Such parameters could provide markers for tumor grading in patients with high-grade glioma (1,2) and for early brain tumor response to antiangiogenic therapy (3). Brain lesions commonly exhibit large regions of spatial heterogeneity or thinly enhancing rims around necrotic cores; in addition, multifocal metastases may be visible throughout the entire brain (4). Effective markers therefore require high-spatiotemporal-resolution whole-brain DCE MRI protocols, as well as accurate and reproducible tracer kinetic parameters (3,5,6).

Many attempts have been made to achieve desired spatial resolution and coverage while preserving rapid sampling of the temporal evolution of contrast agent dynamics. Although direct estimation of tracer-kinetic parameters from undersampled MRI raw data exists (7,8), more commonly, constrained reconstruction algorithms reconstruct the image time series in an intermediate step (9–11) or simultaneously to tracer-kinetic estimation (12). This can be advantageous because it offers great flexibility in the estimation of nuisance parameters, such as the vascular input function (VIF), which is commonly done on the basis of image data.

Extensive efforts by the Radiological Society of North America Quantitative Imaging Biomarkers Alliance DCE MRI task force and other groups have resulted in standardization and characterization of Nyquist-sampled DCE MRI (13). Although much research has been devoted to obtaining 20- to 50-fold undersampled parallel imaging techniques (9–12), a similar characterization has not yet been performed for high-spatiotemporal-resolution whole-brain DCE MRI systems with sparse sampling.

Characterization of measurement uncertainty relies on reproducibility studies in controlled test-retest settings. These tend to be time consuming, resource demanding, and ethically challenging to justify. Recruitment of patients with high-grade glioma is further complicated by disease severity, which lowers willingness of outpatients to accept the inconvenience of non–standard-of-care retest DCE MRI examinations. This unwillingness prevents the full development and characterization of candidate markers for individualized therapy and clinical trials (3,14,15). To determine the intrinsic method uncertainty and hence sample size required for larger clinical trials (15), the use of clinical posttreatment data could be an alternative to obtaining true test-retest data. By setting the focus on stable tissue types with varying parameter magnitudes, worst-case estimates for parameter uncertainty in the target brain tumor tissue could be inferred.

In this work, we demonstrate a fully automated, high-spatiotemporal-resolution, whole-brain DCE MRI pipeline with 30-fold sparse undersampling and no user interaction required. We estimate the reproducibility of this proposed pipeline on the basis of reference regions of stable tissue types during multiple posttreatment time points in patients with brain tumors.

## Materials and Methods

### Participants

In a retrospective study, we estimated reproducibility of the proposed pipeline among participants with high-grade glioma who underwent three consecutive standard-of-care examinations (mean interval between examinations, 64 days; range, 35–231 days). All data were acquired with a single clinical 3.0-T MRI machine (MR750, GE Healthcare) with a 12-channel head-neck-spine receiver coil between August 2016 and April 2019 at the Seaman Family MR Research Centre, Foothills Medical Centre (Calgary, Alberta, Canada). Participants were recruited from the Tom Baker Cancer Centre and provided written informed consent. Data were acquired under a protocol approved by the relevant local institutional review board.

**Figure 1:**
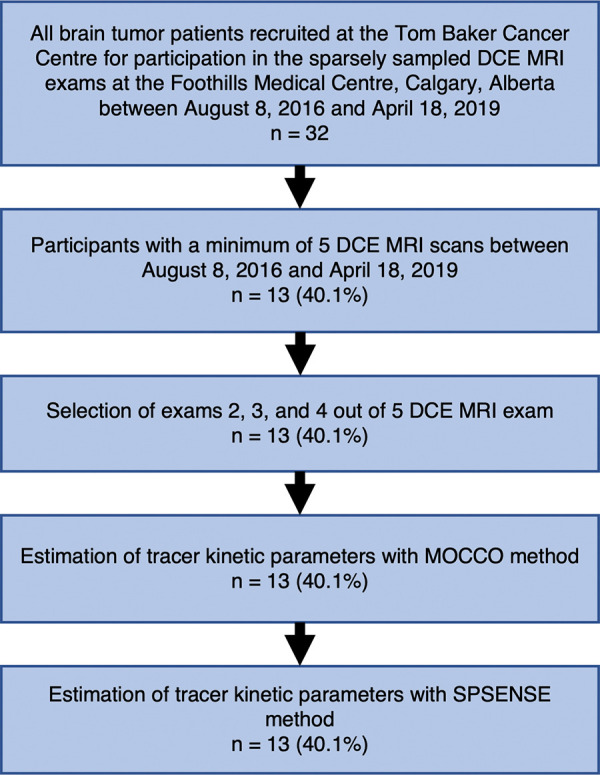
Flowchart of participants and exclusion criteria. Between August 2016 and April 2019, a total of 32 patients with brain tumors were recruited for sparsely sampled dynamic contrast-enhanced (DCE) MRI protocol. After exclusion of all participants with fewer than five DCE MRI examinations in this time period, data from second, third, and fourth DCE MRI examinations were used for reconstruction with model consistency constrained (MOCCO) and sparse sensitivity encoding (SPSENSE) methods.

Participants were selected from an ongoing imaging study of high-grade gliomas. The current study included the first 13 participants with a minimum of five longitudinal MRI examinations among a total cohort of 32 participants; the second through fourth examinations were used in the current study. Participants with fewer than five MRI examinations were excluded. Figure 1 shows a flowchart of participant recruitment and exclusion criteria. Participants received heterogenous and often-changing pharmacologic therapy during the three time points. Medications included dexamethasone, temozolomide, and clobazam.

### Sparse DCE MRI Data Acquisition

**Figure 2:**
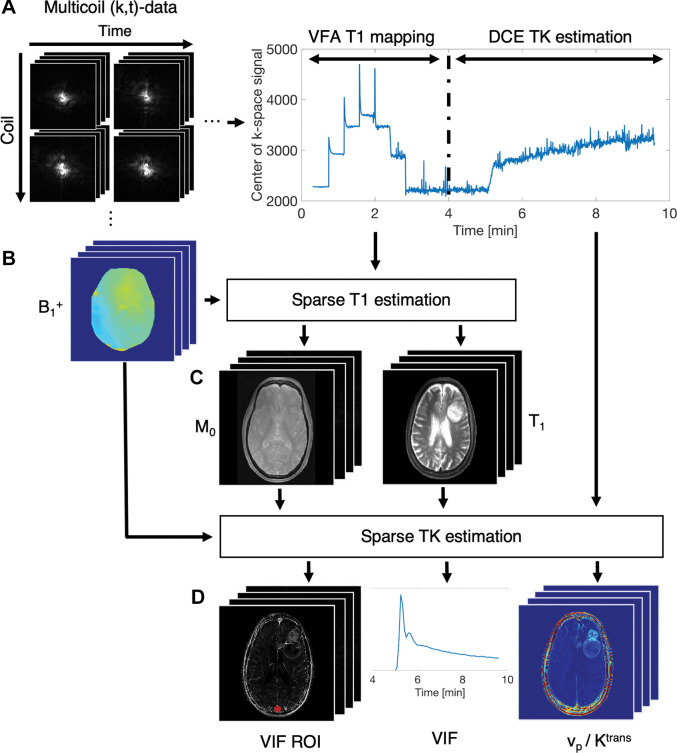
Image shows schematic of pipeline. *A,* Three-dimensional k-space data are continuously acquired over time window of approximately 10 minutes (cubic 240-mm^3^ field of view with voxel size of 0.94 × 1.0 × 2.0 mm^3^, echo time of 1.9 msec, and repetition time of 5 msec). During first part of examination, flip angle is successively ramped up from 1.5° to 15° in seven logarithmically spaced steps to yield variable-flip-angle (VFA) measurements for high-resolution whole-brain T1 mapping. VFA data are used with, *B,* calibration B_1_+ map to generate, *C,* T1 and M_0_ maps before administration of contrast material. *D,* Vascular input function (VIF) regions of interest (ROIs), VIF, and tracer kinetic (TK) parameter maps for plasma volume fraction (v_p_) and volume transfer constant (K^trans^) are estimated from second part of k-space data (in *A*), which monitors contrast enhancement at 5-second temporal resolution. DCE = dynamic contrast enhanced.

Three-dimensional k-space data were continuously acquired during a time window of 9.6 minutes (field of view, 240 × 240 × 240 mm^3^; voxel size, 0.94 × 1.0 × 2.0 mm^3^; echo time msec/repetition time msec, 1.9/5). During this time window, the contrast agent (Gadovist, Bayer AG) at a dose of 0.1 mL/kg body weight was intravenously injected. The readout dimension was fully sampled while the phase encode dimensions were acquired along rasterized spiral-in trajectories. The B_1_^+^ maps were estimated by the vendor-provided Bloch-Siegert method (16). During the first 170 seconds of the MRI examination, the flip angle was successively ramped up from 1.5° to 15° in seven logarithmically spaced steps to yield variable flip-angle measurements for high-spatiotemporal-resolution whole-brain T1 mapping (17). The variable flip-angle data were used with a calibration B_1_^+^ map to generate precontrast-agent T1 and M_0_ maps. The last part of the k-space data after bolus arrival was binned to 5-second temporal resolution to monitor contrast enhancement and allow for estimation of vascular input functions and tracer kinetic parameter maps. Figure 2 illustrates data acquisition and reconstruction steps.

### Sparse DCE-MRI Reconstruction Methods

Lebel et al (10) demonstrated reconstruction of high-spatial-resolution whole-brain DCE MRI time series from highly undersampled raw k-space data for tracer-kinetic parameter estimation. Guo et al (12) demonstrated joint estimation of tracer-kinetic parameter maps and patient-specific VIF. In the current study, we extended the sparse SENSE (SPSENSE) framework by Lebel et al (10) and the model consistency constrained (MOCCO) method by Guo et al (12) with an automated delineation of brain vessels based on common image/time series features in the literature (ie, time to peak, full width at 80% maximum, vesselness [tubularity of the spatial structure], and enhancement relative to period before administration of contrast agent) (this step was performed by Y.B., an MR scientist with 4 years of experience in brain DCE MRI) (18–21). We chose the largest connected vessel as a VIF region of interest (ROI). We then trimmed the outermost voxels to remove possible partial volume-averaging effects. The final VIF was jointly estimated (by Y.B.) from the magnitude of image voxels within the VIF ROI (22). Baseline proton density and T1 maps were estimated at matching spatial resolution and coverage from sparsely sampled B_1_-corrected variable flip-angle T1 mapping (this step was performed by R.M.L., an MR scientist with 10 years of experience in brain DCE MRI) (17). For conversion of image intensity to concentration-time curves, we assumed the fast exchange limit approximation with fixed relaxivity (*r*_1_ = 4.5 mM^–1^ s^–1^) and hematocrit value (45%). Concentration-time curves are assumed to follow the Patlak or extended Tofts-Kety model (23,24), where the appropriate tracer kinetic model was chosen on the basis of the Akaike information criterion (this step was performed by Y.B.) (25,26). To account for bolus transit delay, we fit each voxel with three VIFs: one as extracted and two other functions shifted by plus and minus one time bin. For each voxel, the parameters of the best fit were retained.

The source code for data reconstruction and analysis can be found on GitHub (*https://github.com/usc-mrel/dcemri_pseudo_test_retest;* revision b2b3e4b).

### Statistical Analysis

To determine reproducibility of VIF estimation, we determined the within-case coefficient of variation (COV) (27,28) and reproducibility coefficient (RC) (27) of relevant VIF features (this step was performed by Y.B.) (28–30). Specifically, we determined *(a)* peak concentration and *(b)* area under the curve, both of which are crucial for accurate tracer-kinetic estimation, and *(c)* area under the first pass, which is important for cerebral blood flow measurement.

The advent of high-spatial-resolution DCE MRI protocols has led to increased interest in histogram analyses and histogram-derived statistics to analyze tumor heterogeneity (31). To determine reproducibility of histogram analysis of tracer kinetic parameters, ROIs were manually drawn on five different enhancing tissue types: normal-appearing white matter, the mucosal surface of the nasal mucosa, the choroid plexus, scalp fat, and the temporalis muscle (performed by Y.B. and J.A., a board-certified neuroradiologist with 9 years of experience in head, neck, and spine imaging). The ROIs were not drawn on tumor tissue because of postresection ambiguity and possible tumor progression.

To determine reproducibility of potential markers and the underlying histograms for tracer-kinetic parameters—plasma volume fraction (v_p_) and volume transfer constant (K^trans^)—we computed the COV and RC for the robust histogram statistics of median and 95th percentile (this step was performed by Y.B.) (13,15). Statistical analysis was performed in Matlab, version R2018a (MathWorks).

## Results

### Participant Characteristics

We estimated reproducibility of the proposed pipeline using the DCE MRI examinations of 13 participants with high-grade glioma (mean age ± standard deviation, 61 years ± 10; nine women). Table E1 (online) summarizes participant demographic characteristics, tumor types, and time intervals between successive examinations. A flowchart illustrating recruitment of 32 patients with brain tumors and the criteria for exclusion of 19 participants from this study is shown in Figure 1.

### DCE MRI Examination

**Figure 3:**
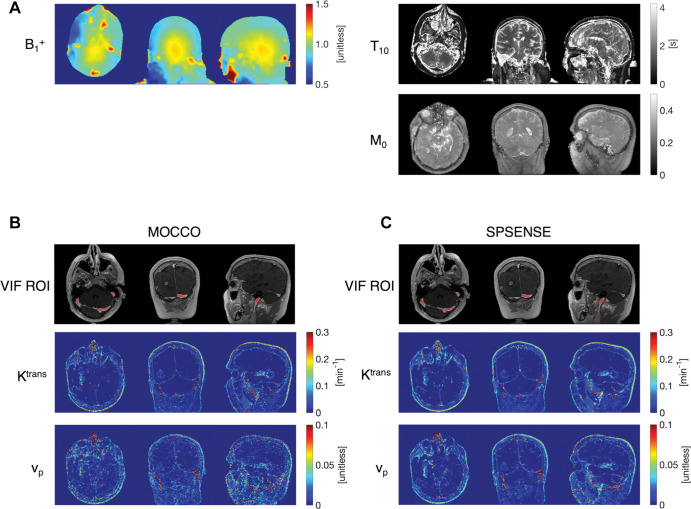
Representative parameter maps of dynamic contrast-enhanced MRI examination in a 59-year-old woman in axial, coronal, and sagittal views for model consistency constrained (MOCCO) and sparse sensitivity encoding (SPSENSE) frameworks. *A,* Baseline maps for radiofrequency inhomogeneity (B_1_+), baseline T1, and proton density map M_0_. *B, C,* Images obtained after administration of contrast agent with regions of interest for vascular input functions on internal jugular veins, transverse and sigmoid dural venous sinuses (red), plasma volume fraction (v_p_), and volume transfer constant (K^trans^) for MOCCO and SPSENSE, respectively.

Figure 3 illustrates outputs of the fully automated DCE MRI pipeline for one representative tumor case in a 59-year-old woman. It includes spatial maps of B_1_^+^, precontrast-agent T1, and proton density M_0_ maps; VIF ROI location; and tracer-kinetic parameters. The B_1_^+^, T1, and M_0_ maps are common to both methods. MOCCO and SPSENSE estimation approaches differ in VIF estimation and in estimated parameter maps for v_p_ and K^trans^. Using both approaches, the internal jugular veins, transverse, and sigmoid dural venous sinuses were detected.

### Reproducibility of Patient-specific VIF Estimation

**Figure 4:**
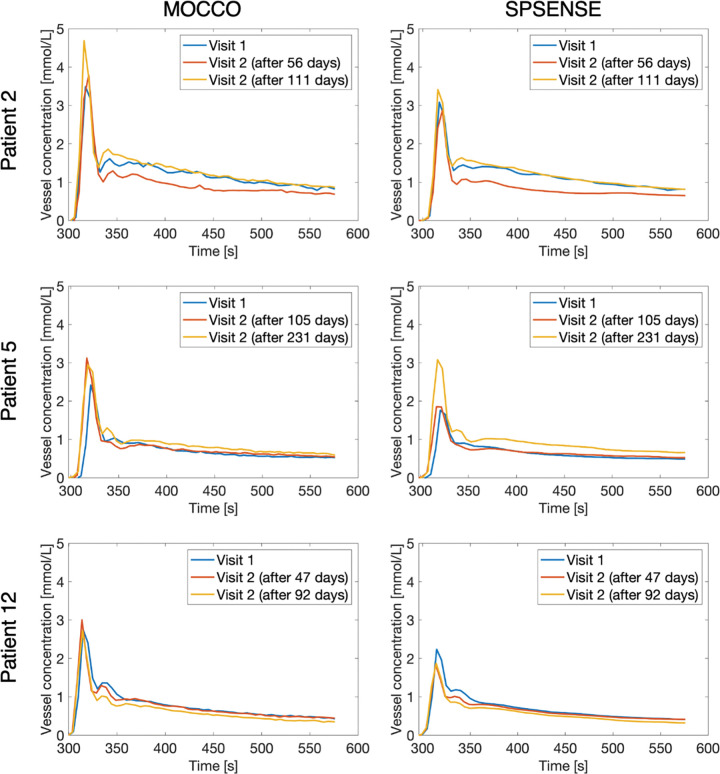
Graphs show representative vascular input functions (VIFs) for different participants, visits, and methods. Each row corresponds to a different participant. Left column shows model consistency constrained (MOCCO) reconstruction, and right column shows VIFs estimated from sparse sensitivity encoded (SPSENSE) image time series. All VIFs show blood concentration.

Figure 4 shows representative VIFs for three participants at three visits each. The VIFs are measured with MOCCO and derived from SPSENSE image time series data. Table 1 lists mean, COV, and reproducibility coefficients for peak concentration, areas under the curve, and areas under the first pass. VIFs estimated in the MOCCO framework were larger in magnitude than SPSENSE-based VIF estimation, with mean area under the curve of 247.5 mM for MOCCO versus 209.6 mM for SPSENSE. The COVs for VIF features were 0.18 for MOCCO and 0.13 for SPSENSE, indicating similar reproducibility of MOCCO and SPSENSE in their ability to measure VIFs. As shown in Figure 4 and Table 1, estimation of VIFs based on the MOCCO method led to overall larger initial enhancement peaks of the VIF, with mean peak concentration of 3.1 mM compared with 2.1 mM for SPSENSE.

**Table 1:**
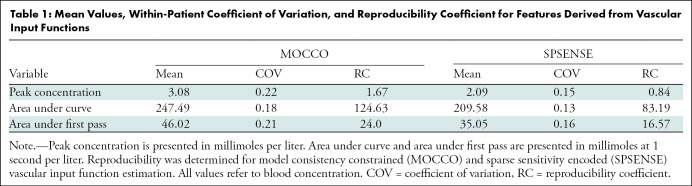
Mean Values, Within-Patient Coefficient of Variation, and Reproducibility Coefficient for Features Derived from Vascular Input Functions

### ROI Placement

**Figure 5:**
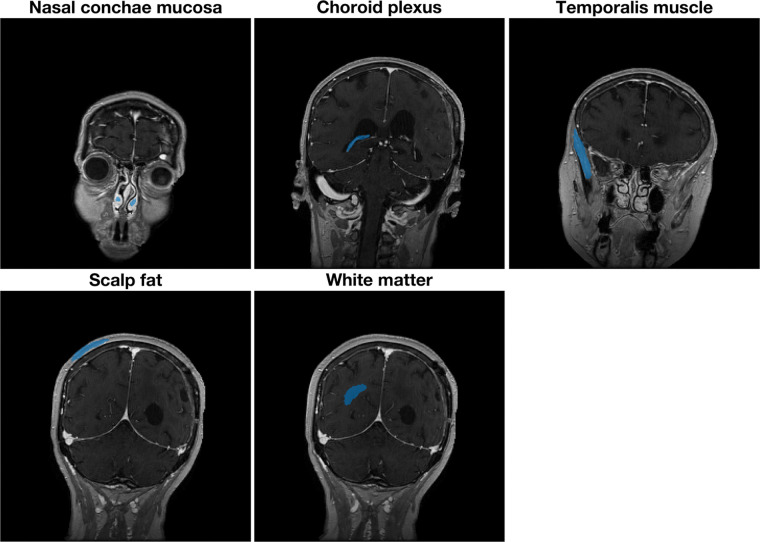
Illustration of three-dimensional regions of interest used to evaluate reproducibility of tracer kinetic parameter estimation. For each region of interest, one representative section of three sections is shown. All regions of interest with exception of nasal mucosa are drawn contralateral to lesion.

Figure 5 illustrates all five three-dimensional ROIs used to compute histogram statistics and to assess their reproducibility. With the exception of the nasal mucosa, all ROIs are drawn contralateral to the lesion. All ROIs spanned at least three sagittal sections. To illustrate the number of data points composing the individual histograms for each ROI, the mean and standard deviation of the ROI sizes for each tissue type are listed in Table 2.

**Table 2:**
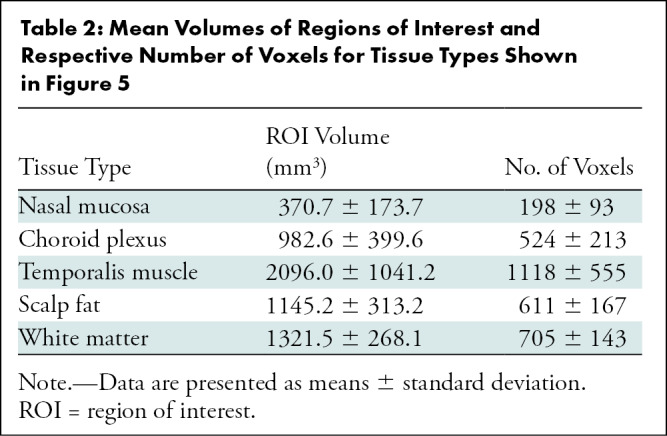
Mean Volumes of Regions of Interest and Respective Number of Voxels for Tissue Types Shown in Figure 5

### Reproducibility of v_p_ Estimation

**Figure 6:**
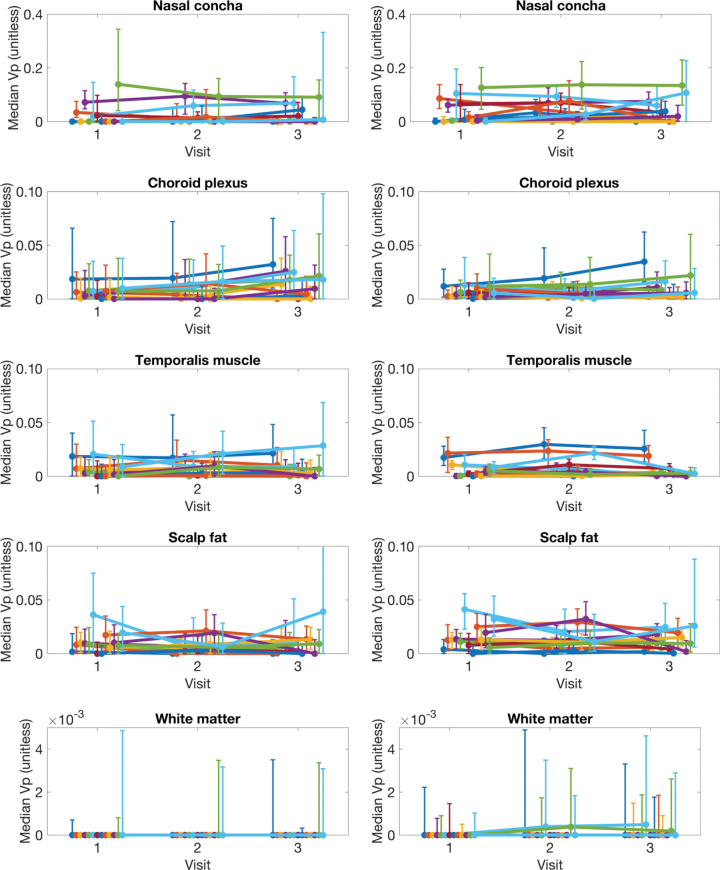
Graphs show temporal evolution of median plasma volume fraction (v_p_) in five regions of interest shown in Figure 5 for different lesions and visits. Each row corresponds to different tissue. Left column shows results for model consistency constrained reconstruction, and right column shows sparse sensitivity encoded reconstruction. Each line corresponds to different participant. Error bars show interquartile range (25th to 75th percentile) for each participant, visit, and tissue region of interest.

Figure 6 shows the temporal evolution of median for v_p_ per region as estimated by MOCCO and SPSENSE. Table 3 lists the mean, COV, and RC for the histogram statistics median and 95th percentile for v_p_. As illustrated in Figure 6, both methods led to similar temporal behavior for median v_p_ across the three visits for the three largest ROIs (ie, temporalis muscle, scalp fat, and white matter). Intrapatient variation across time was similar to interpatient variation across different participants. MOCCO achieved COVs of 87%–117% for median v_p_ in healthy tissue, whereas SPSENSE achieved COVs of 53%–95% for median v_p_. RCs for the median v_p_ are below 0.04 and 0.07 for MOCCO and SPSENSE, respectively. This maximum was achieved in the most strongly vascularized nasal mucosa.

**Table 3:**
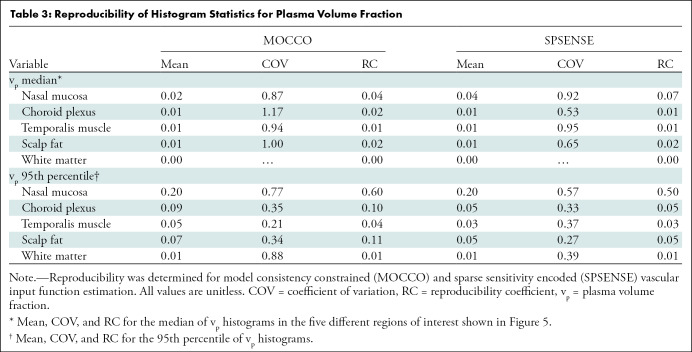
Reproducibility of Histogram Statistics for Plasma Volume Fraction

### Reproducibility of K^trans^ Estimation

**Figure 7:**
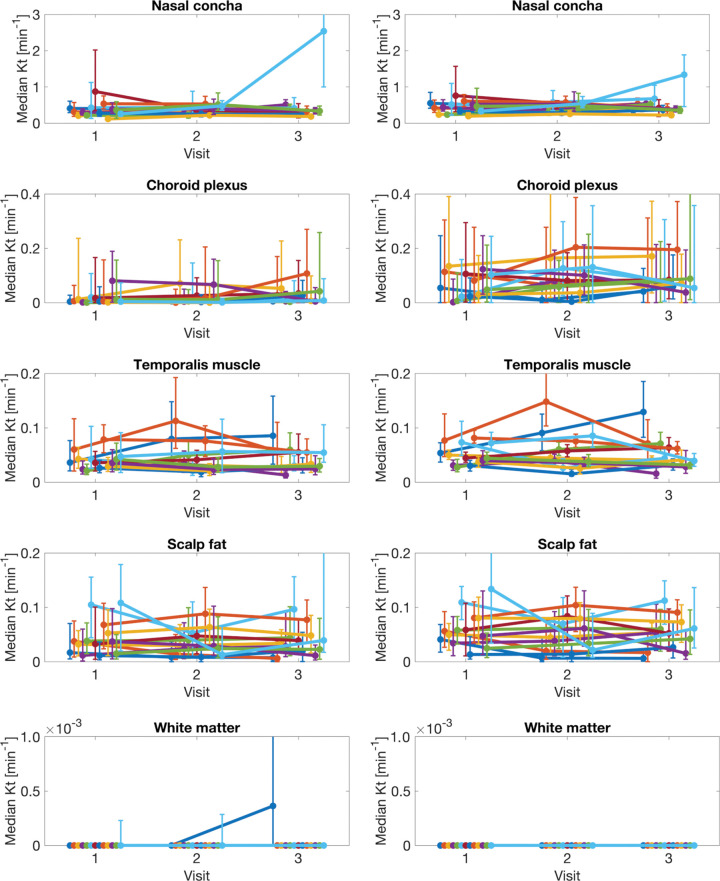
Graphs show evolution of median volume transfer constant (K^trans^) in five regions of interest shown in Figure 5 for different lesions and visits. Each row corresponds to different tissue. Left column shows results for model consistency constrained reconstruction, and right column shows results for sparse sensitivity encoded reconstruction. Each line corresponds to different participant. Error bars show interquartile range (25th to 75th percentile) for each participant, visit, and tissue region of interest.

Figure 7 shows the temporal evolution of median for K^trans^ per region as estimated by MOCCO and SPSENSE. Table 4 lists the mean, COV, and RC for the histogram statistics of median and 95th percentile for K^trans^. In nasal mucosa, temporalis muscle, and scalp fat, the median K^trans^ values estimated by the SPSENSE method (0.46 min^–1^, 0.05 min^–1^, and 0.05 min^–1^, respectively) were on average higher than those estimated by MOCCO (0.41 min^–1^, 0.04 min^–1^, and 0.38 min^–1^, respectively), which is in alignment with lower peak VIF amplitude of SPSENSE. MOCCO achieved COVs of 31%–91% for median K^trans^ in healthy tissue. SPSENSE achieved COVs of 29%–57% for median K^trans^. For the two largest tissue ROIs with nonnegligible K^trans^ (ie, temporalis muscle and scalp fat), both methods estimated median K^trans^ with COVs of 31%–50%, and the higher valued 95th percentile with COVs of 22%–32%. For thinner structures, such as the choroid plexus, or measurements of high permeability, such as the 95th percentile of K^trans^ in the nasal mucosa, the COV was as high as 60%–91%. Similarly, RC estimates increased with increased parameter magnitude or tenuity of the underlying tissue structure. With use of the SPSENSE method, the protocol estimated median K^trans^ in healthy tissues with a maximum RC of 0.06 min^–1^ in large, low-enhancing tissue regions, such as muscle and fat. The maximum reproducibility coefficient for the median K^trans^ was, however, as high as 0.48 min^–1^ in smaller or more strongly enhancing tissue regions, such as the choroid plexus or nasal mucosa.

**Table 4:**
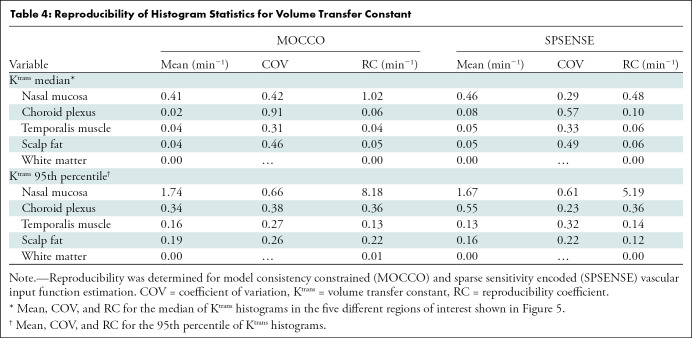
Reproducibility of Histogram Statistics for Volume Transfer Constant

Results of reproducibility for estimation of the extravascular-extracellular volume fraction are shown in Figure E1 and Table E2 (both online).

## Discussion

Advances in sub-Nyquist–sampled dynamic contrast-enhanced (DCE) MRI enables monitoring of brain tumors with millimeter spatial resolution and whole-brain coverage at 5-second temporal resolution. These new features require evaluation regarding achievable test-retest reproducibility in DCE MRI measurements. We estimate that millimeter-resolution whole-brain DCE MRI can be used to estimate the median volume transfer constant in healthy tissues with maximum reproducibility coefficient (RC) of 0.06 min^–1^ in large, low-enhancing tissue regions, such as muscle and fat, and maximum RC less than 0.48 min^–1^ in smaller or more strongly enhancing tissue regions, such as the choroid plexus or nasal mucosa.

We estimate that millimeter-resolution whole-brain DCE MRI can achieve a maximum COV in healthy tissues less than 57% and 91% for median K^trans^ with use of SPSENSE and MOCCO, respectively. In larger, potentially more homogeneous structures (eg, the temporalis muscle or scalp fat), the COV for median K^trans^ is estimated to be 30%–50% for both methods. As a reference, the current Radiological Society of North America Quantitative Imaging Biomarkers Alliance DCE profile specifies an achievable COV of 20% for mean K^trans^ in a tumor ROI of at least 2 cm in diameter, which requires a longitudinal change of 40% in K^trans^ for statistical significance (13). If confirmed in larger cohorts, then the COVs found in this study would imply that even the best method may not yield parameters sensitive enough to monitor subtle longitudinal changes in brain tumors. Differences in estimated COV may be attributed to differences in the use of tracer-kinetic models (which is specified as the standard Tofts model in the profile), estimation of COV in tissues with possibly different permeability strengths (ie, tumor tissue vs healthy tissues) or the time gaps between repeated measurements.

High-spatial-resolution whole-brain DCE MRI guarantees the existence of large-vessel structures inside the field of view. Minimal inflow enhancement and partial volume corruption in the vessel region greatly facilitate automation of VIF estimation. Automation has been a major bottleneck in the standardization of DCE MRI, and the proposed approach helps facilitate automation. We expect that such automation can expedite translation, similar to how fully automated software packages for stroke MRI analysis provide a powerful way to standardize postprocessing and pave the road to clinical deployment (32).

Bias and linearity of DCE MRI cannot be assessed in vivo because of lack of ground-truth values. Therefore, DCE MRI is predominantly suited for longitudinal monitoring, where bias cancels but assessment requires a high degree of known precision (15). We analyzed and characterized reproducibility of the automated DCE MRI method in several different tissues with different enhancement characteristics. Knowledge of the RC of a given method is a crucial first step in determining the detectable effect size in longitudinal studies, such as response to therapy assessment (15).

Our study had several limitations. First, there was a lack of tracer-kinetic parameter ground truth for validation. This limitation is common to most evaluations of DCE MRI pipelines. Estimation of the VIF is a crucial intermediate step and could be cross validated with CT measurements in phantoms (33). For breast DCE MRI, a partial alternative would be to deploy the Stewart-Hamilton theorem to validate the area under the initial peak through independent cardiac output measurements (28). Second, this study used reference regions, not brain tumor tissue. Test-retest studies in sick patients (eg, those with high-grade glioma) and involving procedures with contrast agents are notoriously difficult to conduct because they require patients to appear for additional non–standard-of-care MRI examinations. They are rarely performed despite being an integral part on the path to clinical deployment. Typically, clinical studies are cascaded from pilot studies to randomized clinical trials. For this reason, we pursued a pseudo test-retest study design by selecting tissues that were deemed to be stable during regular treatment follow-up examinations. This relies on the assumption of no or negligible change in the chosen tissues between the time points, which erroneously increases estimated measures of reproducibility if violated. However, because of lack of ground truth and standard reference methods, this assumption may be hard to verify rigorously through nonradiographic measures. As a result of the severity and aggressiveness of the disease, these tissue types do not include the actual tumor target tissue, where possibility of tumor growth and change could not be eliminated. Future research is needed to show whether few representative (healthy) tissue types at various enhancement strength can accurately represent and capture the potential diverse characteristics of reproducibility in lesions as heterogeneous as brain tumors. Finally, our study relied on several simplifying model assumptions. These assumptions include a constant relaxivity and hematocrit value. Hematocrit is known to change throughout the course of treatment (28). We did not model water exchange (34), diffusion of contrast agent (35), or damage to tissue after surgery, which can lead to additional leakage.

We demonstrate a fully automated dynamic contrast-enhanced (DCE) MRI reconstruction and modeling pipeline offering high spatial and temporal resolution with full brain coverage. This includes fast T1 estimation before administration of contrast agents, automated vascular input function extraction, and model-based tracer kinetic parameter mapping. We further demonstrate pseudo test-retest as a possible alternative to assess reproducibility in sick patients. On the basis of the large parameter range for volume transfer constant covered by the various tissue types in this study, we believe that reproducibility in these tissues can give guidance to future study designs involving high-spatiotemporal-resolution DCE MRI.
